# Catecholamines in Post-traumatic Stress Disorder: A Systematic Review and Meta-Analysis

**DOI:** 10.3389/fnmol.2018.00450

**Published:** 2018-12-04

**Authors:** Xiongfeng Pan, Atipatsa C. Kaminga, Shi Wu Wen, Aizhong Liu

**Affiliations:** ^1^Department of Epidemiology and Health Statistics, Xiangya School of Public Health, Central South University, Changsha, China; ^2^Department of Mathematics and Statistics, Mzuzu University, Mzuzu, Malawi; ^3^Department of Obstetrics and Gynaecology and Ottawa Hospital Research Institute, University of Ottawa, Ottawa, ON, Canada

**Keywords:** post-traumatic stress disorder, catecholamines, dopamine, epinephrine, norepinephrine, systematic review, meta-analysis

## Abstract

Studies on the association between post-traumatic stress disorder (PTSD) and levels of catecholamines have yielded inconsistent results. The aim of this study was to conduct a systematic review and meta-analysis to assess whether concentrations of the catecholamines dopamine, norepinephrine, and epinephrine are associated with PTSD. This study searched relevant articles in the following databases: PubMed, Embase, Web of Science, and Psyc-ARTICLES. Each database was searched from its inception to September, 2018. Data related to catecholamine concentrations were extracted for patients with PTSD and the controls to calculate standardized mean differences and to evaluate effect sizes. A meta-analysis was then performed to compare the concentration of each catecholamine between the two groups in blood and/or urine samples. Heterogeneity was quantified using *I*^2^ and its significance was tested using the Q statistics. Subgroup analyses of the types of controls, PTSD assessment tools, and assayed methods used in the studies were performed to explore sources of heterogeneity among studies. Random-effects models were used to combine results from selected studies. A total of 1,388 articles were identified, of which 27 were included in the final analysis. Heterogeneity was high; hence random-effects models were used to combine results of selected studies. Results revealed significantly higher norepinephrine levels in people with PTSD than in the controls [standardized mean difference (SMD) = 0.35, 95% confidence interval (CI): 0.13 to 0.57, *p* = 0.002]. No difference was found in dopamine and epinephrine concentrations between the two groups. Elevated norepinephrine levels may be an important indicator for PTSD.

## Introduction

Post-traumatic stress disorder (PTSD) is a bio-psychosocial dysfunction that can develop in people after they have been exposed to traumatic events, including natural disasters, warfare, traffic accidents, sexual assault, and other threats (McFarlane, 2014; Pollard et al., 2016; Hardy and Mueser, 2017). PTSD includes four symptom clusters: re-experiencing, avoidance, negative alterations in cognition/mood, and alterations in arousal and reactivity (Cordova et al., 2017; Onton et al., 2018). Even worse, PTSD creates more problems than fear memory, and may threaten or damage the quality of life (Burstajn and First, 2014). For instance, patients with PTSD are commonly at a higher risk for intentional self-harm, and suicide (Butterfield et al., 2005). Therefore, PTSD is a major public health problem worldwide, and diagnosing it timely may help to treat or prevent the disease as soon as possible to reduce the burden of the disease.

Several instruments have been used to diagnose PTSD including the diagnostic criteria published in the fifth edition of the Diagnostic, and Statistical Manual of Mental Disorders (DSM-5) and the 11th revision of the International Classification of Diseases (ICD-11) (Hyland et al., 2018). Currently, DSM-5 is the most recommended diagnostic tool to clinicians for diagnosing PTSD. Nonetheless, based on recent advances made in cognitive neuroscience, biomarkers such as catecholamines, cortisol, interleukin 6, and interferon-γ measured in the saliva, serum, urinary, plasma, and cerebrospinal fluid have become new promising options for the screening, treatment, and prevention of PTSD (Kao et al., 2015; Olff and van Zuiden, 2017; Pan et al., 2018). Catecholamines have been identified as a group of monoaminergic neurotransmitters, which include dopamine, noradrenaline, and adrenaline (Highland et al., 2015). The sympathetic nervous system stimulates the release of the catecholamines epinephrine, norepinephrine, and dopamine to mediate adaptive responses to acute stressors. They are also linked with long-term memory of events that induce strong emotions such as fear (Ouyang et al., 2012). They are known to be important in the modulation of the mechanisms underlying different states of PTSD. In addition, stress-responsive neurotransmitters released during emotional arousal are thought to enhance the consolidation of fear memory (Matthews et al., 2012; McLaughlin et al., 2015). Likewise, preclinical evidence has shown that the consolidation and retrieval of traumatic memories are regulated by an interaction between the noradrenergic, glucocorticoid and dopaminergic systems (Eiden, 2013; Hauer et al., 2014).

The tendency of catecholamines to associate with PTSD brought many hypotheses (Norrholm et al., 2013; Bandelow et al., 2017). Among them, dysfunction of the dopaminergic system is one of the most important hypotheses. Dopamine is involved in the regulation of fear-conditioning in a number of brain areas including the amygdala, nucleus accumbens (NAC), ventral tegmental area (VTA), and medial prefrontal cortex (mPFC) (Johnson et al., 2011; Eiden, 2013; Aubry et al., 2016). Furthermore, hyperresponsiveness in the dopaminergic system is common in individuals who have been exposed to stress, which was associated with PTSD symptoms such as restlessness, nightmares, fear memory, and impulsivity (Pezze and Feldon, 2004). Higher dopaminergic activity may contribute to alterations in memory and other cognitive functions, anhedonia, and hypervigilance, all of which are symptoms observed in PTSD patients (Spivak et al., 1999; Sher et al., 2005).

Moreover, existing data suggest that not only was dopamine found to be associated with PTSD, but also higher levels of norepinephrine and epinephrine were observed in PTSD patients (Lee et al., 2016; Gold et al., 2018).

In general, epinephrine from adrenal medulla is identified as the hallmark of acute stress, which is accompanied by noradrenaline release from the locus coeruleus and allows responses to acute stressors to be translated into long-term PTSD symptoms, via increased localized activation, and regulation of circuits in the central nervous system (CNS) (Geracioti et al., 2008).

A possible explanation for this is the over activation of noradrenaline receptors in the amygdala, hippocampus, hypothalamus, striatum, and prefrontal cortex, which could be associated with the flashbacks, and nightmares frequently experienced by those with persistence of PTSD symptoms (O'Donnell et al., 2004; Lee et al., 2017). Previous studies indicated a higher incidence of PTSD among women than among their male counterparts (Mendoza et al., 2016). However, in biological models of PTSD, it is unclear whether sex-mediated output of catecholamines is affected in PTSD (Murphy et al., 2018). To date the biological mechanisms underlying these differential risk factors remain poorly understood.

Taken together, several recent studies investigating the association between catecholamine levels and PTSD found inconsistent results (Glover et al., 2003; Osuch et al., 2009). In some of those studies, individuals with PTSD had higher levels of catecholamines than individuals without trauma exposure or those exposed to trauma but did not develop PTSD (Lemieux and Coe, 1995; De Bellis et al., 1999; Glover et al., 2003; Young and Breslau, 2004). In fact, those with trauma exposure who did not develop PTSD had lower catecholamine levels than those without trauma exposure, indicating a potential mechanism for resilience (Young and Breslau, 2004). However, other studies found that individuals with PTSD had lower levels of catecholamines than the controls (Murburg et al., 1995; Videlock et al., 2008; Osuch et al., 2009).

To date, there is no meta-analysis which investigated the role of catecholamine levels in PTSD (Eiden, 2013). Therefore, there was need to address these inconsistencies using the method of meta-analysis, which is the gold-standard for data aggregation.

The objective of this study is, therefore, to conduct a comprehensive meta-analysis for the first time on the literature related to the relationship between catecholamine concentrations and PTSD, and to quantify the strength of this relationship.

## Materials and Methods

### Search Strategy and Selection Criteria

This systematic review and meta-analysis followed the Preferred Reporting Items for Systematic Reviews, and Meta-Analyses (PRISMA) guidelines (Moher et al., 2010). We searched four electronic databases: PubMed, Embase, Web of Science, and Psyc-ARTICLES. Online electronic databases were searched until September 2018 for articles published in English. Experienced librarians designed these searches, which used the following keywords: (Catecholamine[Title/Abstract] OR Catecholamines[Title/Abstract] OR Dopamine[Title/Abstract] OR Dopamin[Title/Abstract] OR Epinephrine[Title/Abstract] OR Norepinephrine[Title/Abstract] OR Noradrenaline[Title/Abstract] OR Noradrenalin[Title/Abstract] OR Adrenaline[Title/Abstract] OR Adrenalin[Title/Abstract]) AND (PTSD[Title] OR post-traumatic stress disorder[Title] OR posttraumatic stress disorder[Title]). These search terms were adapted for the other databases, for which the detailed search strategies are shown in the Supplemental Material.

### Eligibility Criteria

Studies were eligible for this systematic review, and meta-analysis if they reported (1) PTSD cases and controls, (2) PTSD diagnostic criteria, and (3) the mean and standard deviation (SD) of catecholamine concentrations or if these were provided by the authors upon request. Studies were excluded if (1) they were review articles, and case reports, (2) they studied PTSD in combination with other mental illnesses, (3) the hypothalamic–pituitary–adrenal (HPA) axis was pharmacologically challenged (e.g., by dexamethasone) before catecholamines measurement, (4) they did not study humans, and (5) they were gray literatures (non-published literatures).

Two reviewers [XP and AC] independently screened articles, and selected eligible studies. In case of disagreement the final decision was made by consultation with a third party [AL].

### Data Abstraction

For the purpose of this meta analysis, two independent investigators [XP and AC] extracted the following information according to the inclusion criteria specified above: (1) name of the first author and publication year; (2) country of the study; (3) sample characteristics: sample size, type of catecholamine studied, mean catecholamine concentration and corresponding standard deviation (mean, SD); (4) PTSD participants: trauma type, mean age and corresponding standard deviation (mean, SD), and gender; (5) PTSD assessment method; (6) catecholamine sample collection and assay methods: inter-assay variation, intra-assay variation, sensitivity, and storage temperatures. All the information was organized using EpiData 3.0, and then recorded into Excel spreadsheets.

### Quality Evaluation

Each eligible study was evaluated based on the three broad perspectives: (1) Selection: representativeness of the sample, adequacy of the case definition, and appropriateness of the selection of the controls, and the definition of the controls; (2) Comparability: whether the subjects in different outcome groups are comparable based on the study design or analysis, and whether confounding factors are controlled; (3) Outcome: appropriateness of the method of ascertainment for outcomes of cases and controls, and appropriateness of ascertainment of outcomes of variables and non-response rate. According to the pre-specified criteria, studies were graded as high, moderate, and low quality based on the scores 7–9, 3–6, and 0–3, respectively. Two investigators [XP and AC] independently assessed and graded the eligible studies. Any inconsistencies between them were resolved by group discussion with a third party. Seven studies were judged to be of high-quality, and three of them received a total score of 8. Furthermore, 19 were of moderate quality, and 1 was of low quality.

### Statistical Analysis

All analyses were carried out in R software (version R i386 3.4.2). First, overall meta-analyses comparing catecholamines concentrations between people with PTSD and the controls were performed. Studies used different methods for measuring catecholamines concentration. Therefore, the standardized mean difference (SMD) was used to assess their effect size, which was calculated as Cohen's d (Higgins et al., 2003). The effect size was considered large when it was > 0.8, moderate when it was between 0.5 and 0.8, and low when it was lower than 0.5. Heterogeneity across studies was assessed using Q statistics and quantified by calculating *I*^2^. The *I*^2^ is presented in percentages with a zero percent (0%) value indicating no observed heterogeneity, and higher values indicating increasing heterogeneity. Generally, heterogeneity is categorized as < 25% (low), 25%~75% (moderate) and > 75% (high) (Higgins et al., 2003). Also,τ^2^ statistic was used to assess the total amount of heterogeneity. In the case of significant high heterogeneity (*I*^2^>50%), the random-effects model was used with a restricted maximum-likelihood estimator to obtain a pooled estimate of the effect sizes in the eligible studies, when *I*^2^ > 50%. Otherwise, the randomfixed-effects model was used. The pooled estimated effect size was reported with the corresponding 95% confidence interval (Higgins et al., 2003).

In addition, sources of heterogeneity related to some characteristics reported in the eligible studies were explored by means of subgroup analyses. Thus, the following subgroups were defined according to the availability of data from the eligible studies: trauma type, control type, sample type, assay method, and storage temperature, etc. However, when the number of included studies in a subgroup analysis was < 2, the statistical efficiency was very low (Higgins et al., 2009). Thus, some studies failed to fulfill the criteria for inclusion in subgroup analyses. Additionally, sensitivity analysis was performed to examine whether the stability of the results was influenced by any single study or a cluster of studies sharing some characteristics. The sensitivity analysis involved repeating the meta-analysis by omitting each study in turn.

Finally, potential publication bias was assessed by examining the symmetry of funnel plots when the number of trials reporting the primary outcomes was 10 or more. This was then verified with the Egger's linear regression test (Egger et al., 1997). In all analyses, the level of significance for the effect size estimation was set at the 5%, and all tests were two-sided.

## Results

### Literature Search

**Figure 1:**
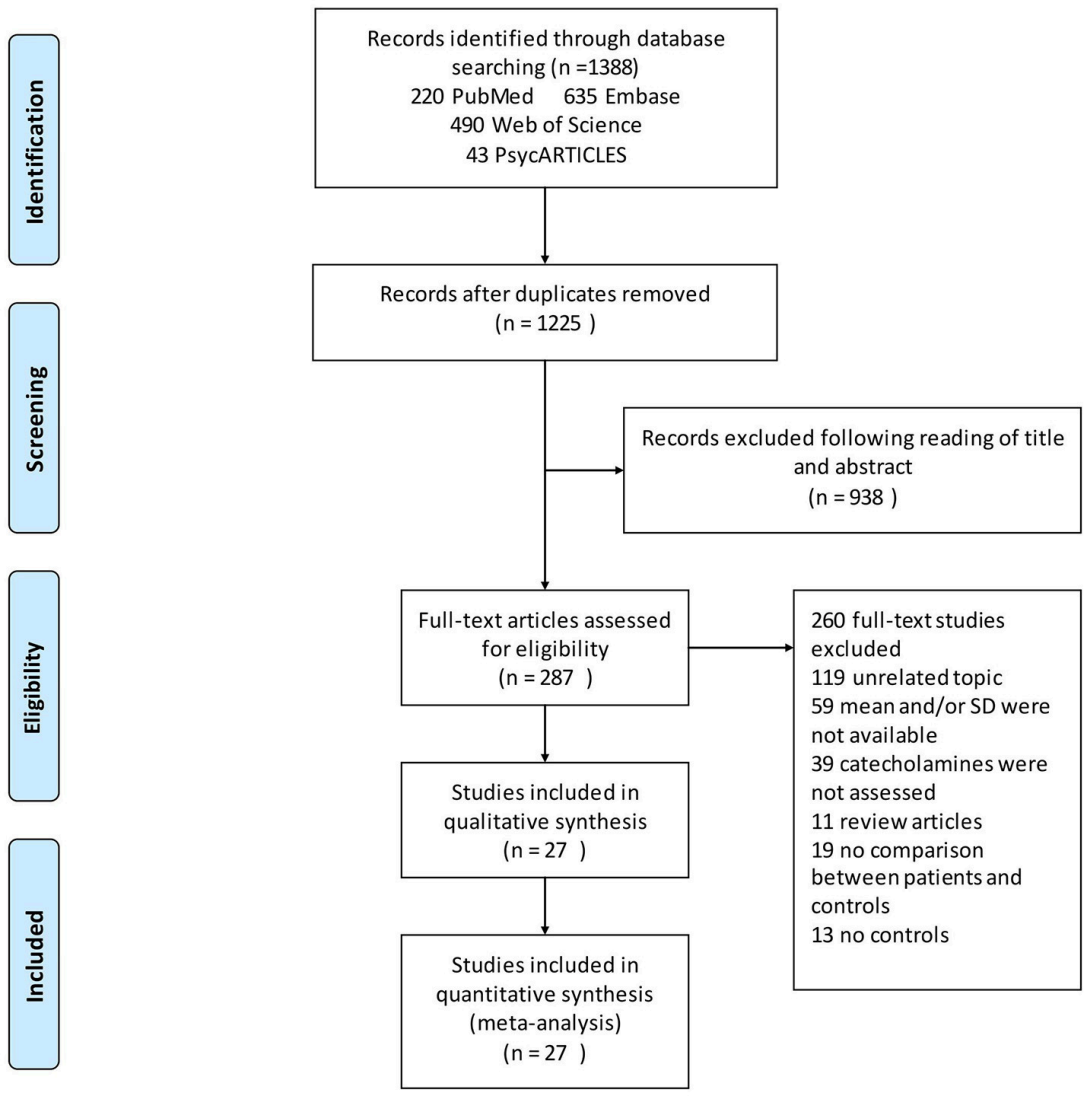
Flowchart of study selection. Showing the process by which relevant studies retrieved from the databases were assessed and selected, or excluded. Preferred reporting items for systematic reviews and meta-analyses (PRISMA) diagram for study search.

Our search identified a total of 1,388 relevant articles. Of these, 220 were from PubMed, 635 from Embase, 490 from Web of Science, and 43 from PsycARTICLES. The 163 duplicated reports were deleted. Further assessment of the 1,225 abstracts resulted in the exclusion of 938 studies that failed to meet the inclusion criteria. The remaining 287 full text articles were then reviewed independently by two authors [XP and AC], which has resulted in the exclusion of further articles. Altogether 59 articles were excluded for not reporting means (SDs), which included studies, whose authors have not replied to the e-mail request before this submission. Another 119 articles were excluded for having unrelated topics, 39 articles for not assessing catecholamines, 11 articles because they were review articles, 19 articles for not comparing patients and controls, and 13 articles for not reporting results for controls. A total of 27 articles that met the inclusion criteria were included in the final analyses (Figure 1).

### Characteristics of Eligible Studies

Most articles reported the following characteristics: trauma type, control type, PTSD participants: trauma type, mean, and SD (mean, SD) of age of participants, sex distribution of participants, time of catecholamine collection, catecholamine collection and assay methods, inter-assay variation, intra-assay variation, sensitivity, and storage temperature. Table 1 gives an overview of the parameters reported in the 27 eligible studies.

**Table 1:** Characteristics of studies included in meta-analysis of catecholamines in post-traumatic stress disorder.

**Study**	**Sample**	**Hormone**	**Country**	**Trauma type**	**Controls**	**Female**	**Mean age**	**PTSD assessment**	**Collection time**		**Method**	**IEV**	**IAV**	**Frozen**	**Quality**
Glover and Poland, 2002	Urinary	DA	USA	Cancer	TC	14(100.0%)	42.0 ± 6.1	DSM-IV	24 h		RIA	10.30%	6.70%	−80°C	6
De Bellis et al., 1994	Urinary	NE/EPI/DA	USA	Abuse	NTC	12(100.0%)	11.5 ± 2.4	DSM-III-R	24 h		GC-MS	5.20%	6.30%	−70°C	8
De Bellis et al., 1999	Urinary	NE/EPI/DA	USA	Abuse	NTC	8(44.4%)	10.4 ± 1.4	DSM-IV	24 h		HPLC	3.00%	5.00%	−80°C	7
Lemieux and Coe, 1995	Urinary	NE/EPI/DA	USA	Combat	TC	11(100.0%)	35.3 ± 6.3	DSM-III-R	24 h		HPLC	6.10%	3.80%	NR	5
Osuch et al., 2009	Urinary	NE/EPI/DA	Canada	•Mixed	TC	8(88.9%)	41.4 ± 12.3	DSM-IV(CAPS)	24 h		HPLC	NR	NR	NR	5
				•trauma											
Spivak et al., 1999	Urinary	NE/EPI/DA	Israel	Combat	NTC	0(0%)	33.1 ± 7.4	DSM-III-R	24 h		HPLC	5.00%	5.00%	−20°C	7
Wingenfeid et al., 2015	Urinary	NE/EPI/DA	USA	•Mixed	NTC	25(12.6%)	57.4 ± 11.0	DSM-IV(CAPS)	24 h		HPLC	NR	NR	0°C	5
				•trauma											
Delahanty et al., 2000	Urinary	EPI	USA	Accident	NTC	36(36.4%)	37.3 ± 17.5	DSM-IV	15 h		HPLC	NR	NR	0°C	4
Murburg et al., 1995	Plasma	EPI	USA	Combat	NTC	0(0%)	42.2 ± 3.7	DSM-III-R	PM	16:00	SIEA	6.50%	5.00%	−70°C	7
Vidović et al., 2011	Plasma	EPI	Croatia	Combat	NTC	0(0.0%)	42.5 ± 6.3	DSM-IV(CAPS)	PM	9:00	HPLC	8.00%	9.00%	NR	5
Yahyavi et al., 2015	Plasma	NE/EPI	Iran	Combat	TC	0(0%)	47.0 ± 5.7	DSM-IV-TR	PM	16:00	ELISA	NR	NR	0°C	6
Chung et al., 2008	Plasma	NE/EPI	USA	Combat	NTC	0(0%)	51.1 ± 2.5	DSM-IV(CAPS)	AM	NR	HPLC	NR	NR	NR	4
Rhind et al., 2017	Plasma	NE/EPI	Canada	Combat	TC	0(0%)	36.3 ± 5.6	DSM-IV(CAPS)	PM	NR	RIA	NR	NR	NR	5
Glover and Poland, 2002	Urinary	NE/EPI	USA	Cancer	TC	14(100.0%)	42.0 ± 7.5	DSM-IV	24 h		NR	NR	NR	NR	4
Jensen et al., 1997	Plasma	NE/EPI	USA	Combat	TC	2(28.6%)	35.0 ± 8.0	DSM-III-R	AM	9:00	RIA	NR	NR	NR	3
Liberzon et al., 1999	Plasma	NE/EPI	USA	Combat	TC	0(0%)	46.4 ± 3.2	DSM-III-R	AM	NR	HPLC	3.00%	5.00%	0°C	8
McFall et al., 1990	Plasma	NE/EPI	USA	Combat	TC	0(0%)	40.5 ± 2.1	DSM-III-R	PM	17:30	SIEA	6.50%	5.00%	−70°C	8
McFall et al., 1992	Plasma	NE/EPI	USA	Combat	TC	0(0%)	40.3 ± 2.3	DSM-III-R	PM	17:30	SIEA	6.50%	5.00%	−70°C	7
Mellman et al., 1995	Urinary	NE	USA	Combat	NTC	0(0%)	40.3 ± 3.6	DSM-III-R	24 h		HPLC	NR	NR	−70°C	5
Geracioti et al., 2008	Plasma	NE	USA	Combat	NTC	0(0%)	41.0 ± 9.0	DSM-III-R	24 h		HPLC	5.90%	8.00%	−80°C	6
Lemieux et al., 2008	Urinary	NE	USA	Abuse	NTC	12(100.0%)	30.3 ± 6.4	DSM-III-R	24 h		HPLC	NR	NR	NR	4
Videlock et al., 2008	Plasma/Urinary	NE	Israel	•Mixed	TC	15(49.0%)	31.2 ± 11.6	DSM-IV(CAPS)	24 h		HPLC	NR	NR	NR	4
				•trauma											
von Kaenel et al., 2010	Plasma	NE	USA	•Mixed	TC	4(27.0%)	58.3 ± 7.4	DSM-IV(CAPS)	PM	12:30	HPLC	1.30%	1.60%	NR	5
				•trauma											
Bierer et al., 2006	Urinary	NE	USA	Attacks	TC	19(59.3%)	42.1 ± 10.1	DSM-IV	24 h		HPLC	NR	NR	NR	5
Yatham et al., 1996	Plasma	NE	Canada	Combat	NTC	10(62.5%)	25.5 ± 12.4	DSM-III-R	AM	20:30	HPLC	6.80%	10.80%	−70°C	6
Yehuda et al., 1998	Plasma	NE	USA	Combat	NTC	0(0%)	43.9 ± 2.6	DSM-III-R	AM	8:00	HPLC	13.00%	5.20%	NR	4
Zhang et al., 2014	Plasma	NE	China	•Mixed	TC	11 (73.3%)	43.0 ± 10.0	DSM-IV(PCL-C)	AM	8:00	ELISA	NR	NR	−70°C	6
				•trauma											

*Characteristics of eligible studies are show in each independent case, reported the following characteristics: trauma type, control type, PTSD participants: trauma type, mean, and SD (mean, SD) of age of participants, sex distribution of participants, catecholamines collection time, catecholamines collection, and assay methods, inter-assay variation, intra-assay variation, sensitivity, and storage temperature. TC, trauma-exposed controls; NTC, non-trauma-exposed Controls; RIA, radioimmunoassay; ELISA, enzyme linked immunosorbent assay; HPLC, High performance liquid chromatography-tandem mass spectrometry; CAPS, clinician-administered PTSD scale; NR, not report; USA, United States of America; DSM, Diagnostic, and Statistical Manual of Mental Disorders; Single isotope enzymatic assay, SIEA; Interassay variation, IEV; Intraassay variation, IAV; DA, dopamine; EPI, epinephrine; NE, norepinephrine; 24 h, used 24-h urine sampling as a measure of catecholamines*.

### Overall Comparison

**Figure 2:**
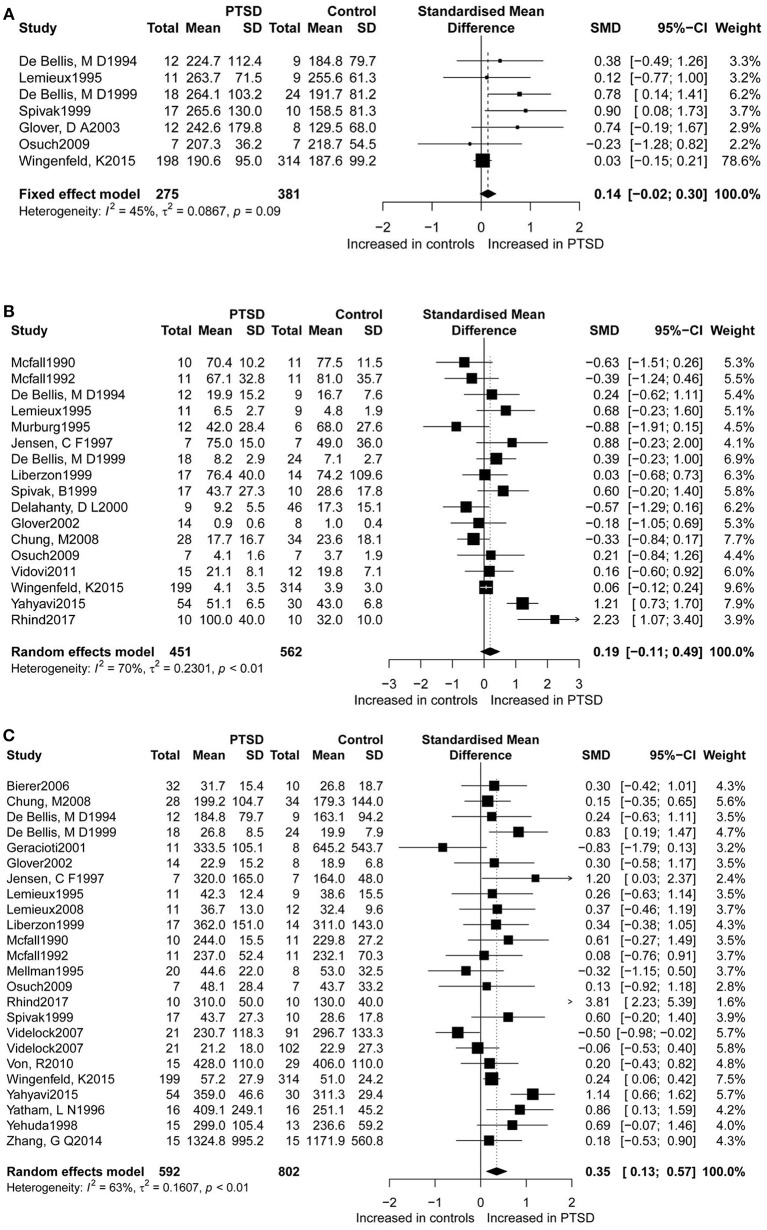
Forest plot of dopamine **(A)**, epinephrine **(B)**, and norepinephrine **(C)** between PTSD participants, and controls. Study effect sizes of catecholamines differences between post-traumatic stress disorder and controls. Each data marker represents a study, and the size of the data marker is proportional to the total number of individuals in that study. The summary effect size for each catecholamine is denoted by a diamond. PTSD, post-traumatic stressdisorder; SMD, standardized mean difference.

Dopamine levels were reported in seven studies (Figure 2A), for which this study found a non-significant difference in dopamine concentrations between PTSD participants and controls. The seven studies accounted for a total of 656 participants (275 PTSD patients and 381 controls), in which dopamine was measured. Epinephrine levels between the two groups were measured in 17 studies (Figure 2B), which corresponded to a total of 1,031 participants (451 PTSD patients, and 562 controls) for epinephrine. Although in these studies higher epinephrine concentrations were observed in PTSD individuals as compared to controls, this result was not significant. The overall analysis comparing concentrations of dopamine (SMD = 0.14; 95%CI [−0.02, 0.30], *P* = 0.088) and epinephrine (SMD = 0.19; 95%CI [−0.11, 0.49], *P* = 0.207) between patients with PTSD and the controls did not indicate any significant differences, but revealed considerable heterogeneity (*I*^2^ = 45.50% and *I*^2^ = 69.70%, respectively).

Furthermore, norepinephrine concentrations were compared in 23 studies between the two groups (Figure 2C). Altogether 592 PTSD patients and 802 controls were included in the studies on norepinephrine levels. Results revealed significantly higher norepinephrine concentrations in people with PTSD than in controls (SMD = 0.35, 95% CI: 0.13 0.57, *p* = 0.002), but with high heterogeneity (*I*^2^ = 62.8%).

### Subgroup Analyses

**Figure 3:**
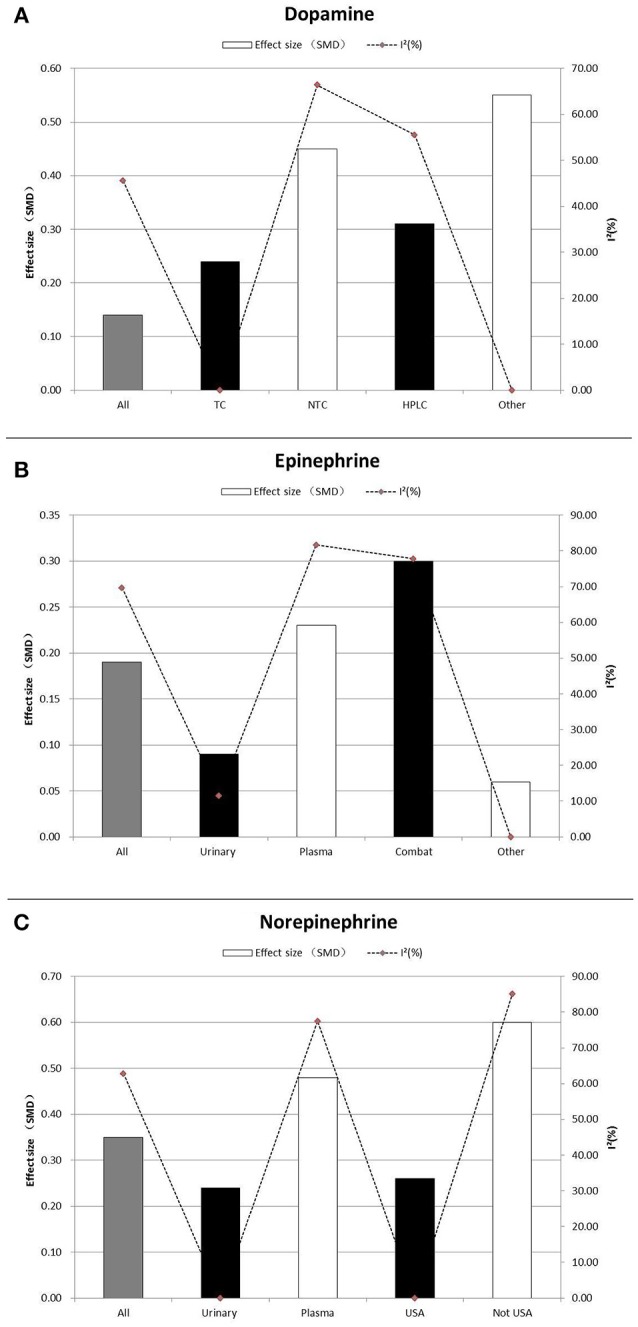
Subgroup Analysis of dopamine **(A)**, epinephrine **(B)**, and norepinephrine **(C)** between PTSD participants, and controls. Study effect sizes of catecholamines differences between post-traumatic stress disorder and controls in the subgroups. Each histogram represents a subgroup, and the height of the histogram is proportional to the total effect size of subgroup in that study. The heterogeneity (*I*^2^) for each subgroup is denoted by a dot with dotted line. SMD, standardized mean difference; TC, trauma-exposed controls; NTC, non-trauma-exposed Controls; HPLC, High performance liquid chromatography-tandem mass spectrometry; USA, United States of America.

Subgroup analyses that assessed dopamine concentrations did not show any significant associations. Similarly, most subgroup analyses comparing epinephrine concentrations between participants with PTSD and the controls led to non-significant differences. Trauma type explained all model heterogeneity with no significant residual heterogeneity (*I*^2^ = 0). On the other hand, sample type significantly explained a large amount of the heterogeneity, but residual heterogeneity (*I*^2^ = 11.5%) was still detected (Table 2 and Figure 3B). Non-significant differences between PTSD and controls were found in relation to gender of the study participants. Concentrations of norepinephrine were higher in urinary samples in participants with PTSD than in the controls (SMD ranging from 0.10 to 0.38, *p* = 0.001), with no heterogeneity (*I*^2^ = 0%). Additionally, for the norepinephrine studies conducted in the United States, the geographical differences significantly explained all heterogeneity (SMD ranging from 0.13 to 0.39, *p* < 0.001, *I* ^2^ = 0%). Non-significant differences between PTSD and the controls were found in the following subgroup analyses: studies conducted outside of the United States (SMD 0.60, 95% CI-0.01 to 1.22; *p* = 0.056), assayed method was HPLC (SMD 0.20,−0.01 to 0.39; *p* = 0.055), and studies without storing sample in cold (SMD 0.38,−0.03 to 0.78; *p* = 0.068) (Table 2 and Figure 3C).

**Table 2:** Subgroup Analysis of dopamine, epinephrine and norepinephrine between PTSD participants and controls.

	***N***	**SMD**	**(95% CI)**		***Z***	***P***	**Heterogeneity**				
							**Q statistic**	**(DF**	**p-value)**	**τ^2^**	**I^2^**
**DOPAMINE**											
All	7	0.14	−0.02	0.30	1.71	0.088	11.00	6	0.0884	0.0867	45.50%
**Controls type**											
TC	3	0.24	−0.31	0.78	0.85	0.396	1.94	2	0.3782	0.0000	0.00%
NTC	4	0.45	−0.06	0.95	1.74	0.081	8.92	3	0.0304	0.1622	66.40%
**PTSD assessment**											
DSM-IV	4	0.30	−0.18	0.78	1.24	0.216	7.15	3	0.0673	0.1306	58.00%
Other	3	0.49	−0.01	0.98	1.93	0.054	1.72	2	0.4229	0.0000	0.00%
**Frozen**											
Yes	4	0.45	−0.06	0.95	1.74	0.081	8.92	3	0.0304	0.1622	66.40%
No	3	0.24	−0.31	0.78	0.85	0.396	1.94	2	0.3782	0.0000	0.00%
**EPINEPHRINE**											
All	17	0.19	−0.11	0.49	1.26	0.207	52.86	16	< 0.0001	0.230	69.70%
**Sample**											
Urinary	8	0.09	−0.06	0.25	1.19	0.235	7.91	7	0.3410	0.013	11.50%
Plasma	9	0.23	−0.37	0.82	0.75	0.453	43.71	8	< 0.0001	0.647	81.70%
**Study country**											
USA	12	0.00	−0.14	0.14	0.03	0.980	16.60	11	0.1201	0.049	33.80%
Not USA	5	0.85	0.22	1.48	2.63	0.009	12.39	4	0.0147	0.337	67.70%
**Trauma type**											
Combat	11	0.30	−0.20	0.80	1.18	0.238	45.06	10	< 0.0001	0.527	77.80%
Other	6	0.06	−0.10	0.21	0.68	0.497	4.51	5	0.4782	0.000	0.00%
**Gender**											
Female	8	0.09	−0.07	0.24	1.13	0.260	8.27	7	0.3095	0.019	15.30%
Male	9	0.21	−0.37	0.78	0.71	0.478	43.21	8	< 0.0001	0.605	81.50%
**Controls type**											
TC	9	0.43	−0.14	0.99	1.47	0.141	32.28	8	< 0.0001	0.541	75.20%
NTC	8	0.03	−0.12	0.18	0.38	0.703	11.33	7	0.1247	0.049	38.20%
**PTSD assessment**											
DSM-IV	9	0.29	−0.14	0.72	1.33	0.184	40.16	8	< 0.0001	0.303	80.10%
Other	8	0.06	−0.24	0.37	0.41	0.683	12.42	7	0.0875	0.155	43.60%
**Assayed methods**											
HPLC	9	0.06	−0.08	0.21	0.82	0.412	10.04	8	0.2622	0.021	20.30%
Other	8	0.30	−0.40	1.00	0.84	0.401	37.20	7	< 0.0001	0.802	81.20%
**Frozen**											
Yes	11	0.06	−0.29	0.41	0.34	0.736	33.95	10	0.0002	0.211	70.50%
No	6	0.55	−0.13	1.23	1.58	0.114	18.38	5	0.0025	0.501	72.80%
**Norepinephrine**											
All	24	0.35	0.13	0.57	3.12	0.002	61.90	23	< 0.0001	0.161	62.80%
**Sample**											
Urinary	11	0.24	0.10	0.38	3.35	0.001	7.64	10	0.6644	0.000	0.00%
Plasma	13	0.48	0.06	0.90	2.24	0.025	53.25	12	< 0.0001	0.433	77.50%
**Study country**											
USA	16	0.26	0.13	0.39	3.91	< 0.0001	14.84	15	0.4632	0.000	0.00%
Not USA	8	0.60	−0.01	1.22	1.91	0.056	46.60	7	< 0.0001	0.627	85.00%
**Trauma type**											
Combat	13	0.55	0.15	0.95	2.67	0.008	40.86	12	< 0.0001	0.364	70.60%
Other	11	0.18	0.05	0.32	2.65	0.008	13.63	10	0.1906	0.027	26.60%
**Gender**											
Female	14	0.22	0.09	0.35	3.28	0.001	19.58	13	0.1061	0.043	33.60%
Male	10	0.50	0.01	1.00	2.00	0.046	38.31	9	< 0.0001	0.461	76.50%
**Controls type**											
TC	14	0.42	0.05	0.80	2.22	0.026	47.14	13	< 0.0001	0.341	72.40%
NTC	10	0.28	0.13	0.42	3.79	0.000	14.72	9	0.0988	0.055	38.90%
**PTSD assessment**											
DSM-IV	12	0.38	0.05	0.71	2.24	0.025	47.27	11	< 0.0001	0.224	76.70%
Other	12	0.35	0.11	0.59	2.82	0.005	14.32	11	0.2159	0.055	23.20%
**Assayed methods**											
HPLC	16	0.20	−0.01	0.39	1.92	0.055	25.54	15	0.0432	0.057	41.30%
Other	8	0.78	0.22	1.34	2.71	0.007	24.81	7	0.0008	0.447	71.80%
**Frozen**											
Yes	13	0.38	0.12	0.65	2.87	0.004	25.76	12	0.0116	0.105	53.40%
No	11	0.38	−0.03	0.78	1.83	0.068	33.97	10	0.0002	0.300	70.60%

*Subgroup analyses are performed to compare the concentration of each catecholamine between the PTSD and the controls. Heterogeneity was quantified using I^2^ and its significance was tested using the Q statistics. PTSD, post-traumatic stress disorder; SMD, standardized mean difference; DF, degrees of Freedom; DSM-IV, Diagnostic, and Statistical Manual of Mental Disorders 4th edition; TC, trauma-exposed controls; NTC, non-trauma-exposed Controls; HPLC, High performance liquid chromatography-tandem mass spectrometry; USA, United States of America*.

After analyzing the pooled data, we performed each analysis separately for data from plasma and urine if the requirements for subgroup analyses were met. Most of the results were similar to those of the subgroup analyses performed using pooled data (Table 3). Nevertheless, one subgroup analysis comparing epinephrine concentrations in urinary samples of PTSD patients to the controls led to a significant difference. Also, we observed significantly higher epinephrine concentrations in PTSD patients compared to the controls (SMD 0.51, 95% CI−0.02 to 1.00; *p* = 0.043), with no heterogeneity (*I*^2^ = 0%). when PTSD was diagnosed using DSM-IV. Additionally, for the epinephrine concentrations in PTSD patients compared to the non-trauma-exposed controls, the types of controls used explained all heterogeneity (*I*^2^ = 0%). We also observed significantly higher urinary norepinephrine concentrations in PTSD patients with non-combat trauma compared to the controls (SMD 0.25, 95% CI 0.10 to 0.40; *p* = 0.001), with no heterogeneity (*I*^2^ = 0%). However, no significant differences between combat trauma-exposed PTSD patients and the controls were observed. Similarly, we observed significantly higher urinary norepinephrine concentrations in PTSD patients compared to non-trauma-exposed controls (SMD 0.30, 95% CI 0.08 to 0.53; *p* = 0.001), but no significant differences between PTSD patients and trauma-exposed controls.

**Table 3:** Subgroup Analysis of epinephrine and norepinephrine in urinary and plasma between PTSD participants and controls.

	**N**	**SMD**	**(95% CI)**		**Z**	***P*-value**	**Heterogeneity**				
							**Q statistic**	**(DF**	***p*-value)**	**τ^2^**	**I^2^**
**EPINEPHRINE-URINARY**											
**Controls type**											
NTC	3	0.23	−0.31	0.77	0.83	0.409	1.80	2	0.4062	0.000	0.00%
TC	5	0.08	−0.08	0.24	0.99	0.320	5.84	4	0.2111	0.037	31.60%
**PTSD assessment**											
DSM-IV	5	0.51	0.02	1.00	2.02	0.043	0.55	4	0.7589	0.000	0.00%
Other	3	0.05	−0.11	0.21	0.59	0.556	4.32	2	0.3640	0.007	7.50%
**EPINEPHRINE-PLASMA**											
**Controls type**											
TC	6	0.52	−0.28	1.32	1.28	0.199	29.15	5	< 0.0001	0.795	82.80%
NTC	3	−0.28	−0.76	0.19	−1.17	0.240	2.65	2	0.2660	0.046	24.50%
**PTSD ASSESSMENT**											
DSM-IV	4	0.75	−0.26	1.75	1.46	0.146	28.00	3	< 0.0001	0.907	89.30%
Other	5	−0.22	−0.74	0.30	−0.83	0.409	6.75	4	0.1497	0.143	40.70%
**ASSAYED METHODS**											
HPLC	3	−0.13	−0.49	0.23	−0.69	0.490	1.39	2	0.4993	0.000	0.00%
Other	6	0.39	−0.53	1.32	0.83	0.405	34.48	5	< 0.0001	1.114	85.50%
**NOREPINEPHRINE-URINARY**											
**Study country**											
USA	8	0.26	0.11	0.42	3.39	0.001	5.09	7	0.6486	0.000	0.00%
Not USA	3	0.11	−0.27	0.49	0.57	0.570	1.99	2	0.3699	0.000	0.00%
**Trauma type**											
Combat	3	0.18	−0.36	0.72	0.66	0.509	2.52	2	0.2830	0.048	20.80%
Other	8	0.25	0.10	0.40	3.28	0.001	5.05	7	0.6540	0.000	0.00%
**Controls type**											
TC	5	0.11	−0.20	0.43	0.70	0.485	1.08	4	0.8974	0.000	0.00%
NTC	6	0.30	0.08	0.53	2.62	0.009	5.75	5	0.3317	0.013	13.00%
**Frozen**											
Yes	6	0.30	0.07	0.52	2.59	0.010	5.70	5	0.3363	0.013	12.30%
No	5	0.13	−0.19	0.44	0.78	0.434	1.26	4	0.8682	0.000	0.00%
**NOREPINEPHRINE-PLASMA**											
**Trauma type**											
Combat	10	0.67	0.18	1.16	2.68	0.007	35.93	9	< 0.0001	0.435	75.00%
Other	3	−0.09	−0.58	0.41	−0.35	0.723	4.08	2	0.1299	0.098	51.00%
**Controls type**											
TC	9	0.61	0.04	1.18	2.11	0.035	44.00	8	< 0.0001	0.584	81.80%
NTC	4	0.26	−0.36	0.89	0.83	0.404	8.97	3	0.0297	0.262	66.60%
**Assayed methods**											
HPLC	7	0.13	−0.28	0.55	0.63	0.528	16.72	6	0.0104	0.192	64.10%
Other	6	0.98	0.26	1.71	2.66	0.008	22.10	5	0.0005	0.596	77.40%

*Results are presented in this Table for studies which met the criteria for the subgroup analysis. Subgroup analysis of epinephrine and norepinephrine in urinary and plasma between PTSD participants and controls. Heterogeneity was quantified using I^2^ and its significance was tested using the Q statistics. PTSD, post-traumatic stress disorder; SMD, standardized mean difference; DF, degrees of Freedom; DSM-IV, Diagnostic, and Statistical Manual of Mental Disorders 4th edition; TC, trauma-exposed controls; NTC, non-trauma-exposed Controls; HPLC, High performance liquid chromatography-tandem mass spectrometry; USA, United States of America*.

### Sensitivity and Bias Analysis

Sensitivity analysis indicated that any single study or a cluster of studies sharing some characteristics had a small influence on the SMD and the corresponding 95% CI. Publication bias was not reported for dopamine because the number of studies reporting dopamine was < 10 for each comparison (Table 2 and Figure 3A) No obvious asymmetry was observed in the shape of the funnel plot, and the Egger's test scored a *p*-value of 0.626 and 0.282 for epinephrine and norepinephrine, respectively, implying the lack of publication bias for studies on these two biomarkers.

## Discussion

To the best of our knowledge, this is the first systematic review and meta-analysis to explore the relationship between the PTSD status and levels of catecholamines (dopamine, epinephrine, and norepinephrine) in the plasma and/or urine. The findings suggest no association of dopamine or epinephrine concentrations with PTSD. However, norepinephrine levels were higher in PTSD patients than in controls. Nonetheless, the heterogeneity was considerably high, indicating that differences between subgroups might be present. Contrary to a previous study (Lee et al., 2017), this meta-analysis did not reveal a significant difference in dopamine concentrations between PTSD participants and controls. For epinephrine, this meta-analysis showed higher concentrations in PTSD individuals than in the controls, which however were not significant. Therefore, more studies are needed to assess if dopamine or epinephrine are truly associated with PTSD (Yahyavi et al., 2015).

In contrast, PTSD patients had significantly higher norepinephrine levels than controls, which is in line with previous hypothesis that predicted an association of norepinephrine with PTSD (Rebecca and Hendrickson, 2016).

It should be noted that in this meta-analysis data of plasma and urine concentrations were pooled for comparing PTSD cases and controls. While this may increase the power of statistical analyses, from a biological point of view, this approach may be limited by potential confounding factors in different types of samples (Dikanovic et al., 2011). Thus, after completing analyses of pooled data, we also performed each analysis separately in data from plasma and urine. Moreover, studies reporting other comorbid mental disorders were excluded from this meta-analysis to focus on the relationship between the catecholamines and PTSD only, although an investigation on these studies could be important. Likewise, studies on catecholamine levels in CSF or metabolites of catecholamines could not be included in the analyses, because either the number of the studies containing this information was is not enough to perform the relevant analyses, or the studies containing this information did not meet eligibility criteria, e.g., they reported comorbidity between anxiety/depression and PTSD cases (Sher et al., 2005).

Although anxiety/depression and PTSD are often comorbid, there was considerable variation in the comorbidity rates of anxiety/depression and PTSD, which may be confounded by variables that were not measured in the population (Koenig et al., 2018; Malgaroli et al., 2018), e.g., the variation could be related to the presence or absence of disturbances in the HPA axis. Nevertheless, changes in catecholamine metabolism or abnormalities in monoaminergic transmitter may contribute to the comorbidity of depression with PTSD (Sher et al., 2005; Strawn et al., 2010). For example, depressed subjects with PTSD had higher homovanillic acid (HVA) levels than the depressed subjects without comorbid PTSD, although the two groups did not differ in 5-hydroxyindolacetic acid (5-HIAA) and 3-methoxy-4-hydroxyphenylglycol (MHPG) levels (Sher et al., 2005). In addition, studies on patients with substance use disorder (SUD) were excluded from this meta-analysis, because they may have taken a variety of substances or received medication that can affect catecholamine release and/or metabolism (Bountress et al., 2017).

Although males generally have a higher risk of experiencing trauma than females, females are twice more likely to develop PTSD than males (Gill et al., 2005). Current studies of individuals with PTSD provide evidence for alterations in the neuroendocrine system, e.g., catecholamines are affected by sex steroid hormones (Bangasser et al., 2018; Cao et al., 2018). In general, women appear to have a more sensitized HPA axis than men, and our findings indicated that females with PTSD showed higher concentrations of norepinephrine than female controls (Bangasser et al., 2016). Such differences were also apparent between males with PTSD and male controls, but with higher heterogeneity. This may explain why women are more vulnerable than men to the development of post-trauma symptoms and take longer than men to recover from them. Moreover, gender-specific psychobiological reactions (particularly of norepinephrine) to trauma may contribute to sex differences for a higher PTSD risk. For example, estrogen can increase norepinephrine in the target regions of the locus coeruleus by enhancing the capacity for norepinephrine synthesis, while reducing norepinephrine degradation, potentially increasing arousal in females (Bangasser et al., 2016). This effect could translate into hyperarousal in women under conditions of norepinephrine hypersecretion that occur in PTSD (Bangasser et al., 2013). However, it is noteworthy that the incidence of various types of trauma is generally also disproportionate between males and females, hence further investigations for confounding factors are necessary (Farhood et al., 2018).

### Subgroup Analyses

Significantly high heterogeneity existed when assessing the relationship of catecholamines with PTSD, suggesting that there might have been some unmeasured moderators. With regard to dopamine, subgroup analyses indicated that the type of controls, PTSD assessment tools and use of refrigeration to preserve samples significantly explained heterogeneity with no significant residual heterogeneity. Also, subgroup analyses for epinephrine indicated that PTSD patients had significantly higher epinephrine concentrations than the controls in countries other than the United States. This result may suggest that different countries or regions may form different homogenous groups when investigating the relationship between catecholamines and PTSD. However, since more studies for this systematic review and meta-analysis were conducted in the United States, more future investigations are needed in other countries to verify this hypothesis Unfortunately, heterogeneity resulting from social political, economic, ethnic and cultural factors, and the level of technical experience could not be evaluated, because these characteristics were rarely reported in the eligible studies. Thus, we considered country of study as a likely substitute because it contains all those characteristics. Nonetheless, since most of the studies were conducted in the United States, grouping according to individual countries would not be very meaningful because other countries had relatively few publications on this topic. Thus, we grouped the studies as “studies conducted in the USA” and “studies conducted in other countries.” This grouping formed the categories of the subgroup “country of study” in this investigation. Additionally, for epinephrine, trauma type explained all model heterogeneity with no significant residual heterogeneity. It is difficult to explain this result, but it might suggest that differences in epinephrine concentrations were related to being exposed to trauma in general rather than manifestation of PTSD (Eiden, 2013). Findings of the current study suggest the need to consider trauma type as an important factor of PTSD (Hodgdon et al., 2018). In agreement with this suggestion, Guina, J et al found that combat trauma was associated with total PTSD severity, arousal and intrusions (especially physical symptoms) (Guina et al., 2018). However, non-combat trauma was associated with conscious avoidance and negative cognitions/mood. To date the biological mechanisms underlying these risk factors remain poorly understood (Guina et al., 2018). According to these results, it may well be that trauma type affects the output of epinephrine in PTSD. Moreover, further studies will be needed to explore potential pharmacological mechanisms or the relationship between epinephrine and PTSD symptoms. Furthermore, sample types significantly explained a large amount of the heterogeneity, but residual heterogeneity was still detected (*I*^2^ = 11.5%). It is known that the epinephrine release follows a circadian rhythm. In this regard sample type (urine or plasma) might influence the results (Glover and Poland, 2002).

Results showed that norepinephrine concentrations were higher in urinary samples in participants with PTSD than in controls, with no heterogeneity. This may imply that in future studies urinary samples should be preferred for norepinephrine testing (Bandelow et al., 2017).

For studies on norepinephrine conducted in the United States, no heterogeneity was detected (SMD ranging from 0.13 to 0.39, *p < * 0.001, *I*^2^ = 0%). This may suggests that the United States had a relatively stable infrastructure for testing norepinephrine levels resulting in reliable outcomes (Rebecca and Hendrickson, 2016).

After analysis of the pooled data, we performed each analysis separately for data from plasma and urine (Table 3). However, the random effect model incorporates the estimate of the between study variance to calculate the pooled SMD, and this estimate is more prone to error when the study included in subgroup analyses were < 2 (Higgins et al., 2009). Thus, some studies failed to be included in subgroup analyses. Contrary to the outcomes of subgroup analyses discussed above, we observed significantly higher epinephrine concentrations in PTSD patients which were diagnosed by DSM-IV, compared to controls. This result may indicate that the diagnostic tools may account for the different results of subgroup analyses, and DSM-IV may produce consistent results when used in different study populations. Thus, compared with other scales and diagnostic criteria, we recommend the use of DSM diagnostic criteria (Schnyder et al., 2015). This criterion has proved to show a high degree of reliability and validity, and can provide a better reference for further studies on PTSD and catecholamines (Hansen et al., 2017).

Also, in this study, significantly higher urinary norepinephrine concentrations were observed in PTSD patients compared to non-trauma-exposed controls, but no significant differences between PTSD patients and trauma-exposed controls were observed. Therefore, exposure to traumatic events may affect norepinephrine levels, without additional independent effects of PTSD. The neurobiological pathways of norepinephrine in acute stress disorder were well-described (Thoma et al., 2012). But the exact biological mechanisms underlying the altered long-term norepinephrine output as a result of trauma remain largely unknown (Beis et al., 2018). Speculatively, increased output after trauma may evolve as a compensatory anti-glucocorticoid mechanism, to inhibit negative effects of a long-term increase in negative glucocorticoid feedback and sensitivity of glucocorticoid receptors that has been observed in trauma-exposed participants irrespective of PTSD status (Nawata et al., 1985; Zenko et al., 2018). However, this remains to be further investigated. This study also found that the type of sample affects heterogeneity in subgroup analyses. For example, heterogeneity in urine samples was significantly lower than that in plasma samples. Especially for norepinephrine in plasma samples, results from the studies included in these subgroup comparisons were highly heterogeneous.

It is difficult to explain this result, but with regard to methodological aspects, due to the pulsatile nature of catecholamines release, there are inherent limitations when using single-point measurements of basal catecholamines levels (Nicolau et al., 1985; Kondo et al., 2002). More specifically, it is known that the timing of the measurements throughout the day is important as catecholamine release follows a circadian rhythm (Rao et al., 1995; James et al., 2014). Although most plasma samples studies reported the specific time of assessments, we cannot exclude that variation in assessment timing affected our results. Thus, the results should be interpreted cautiously. However, a possible explanation for this result is that the 24-h urinary cortisol has been considered to provide an integrated measure that was more reliable and stable than plasma samples.

In spite of showing an association between some catecholamines and PTSD, this meta-analysis is subject to several limitations.

First, the meta-analysis for dopamine and epinephrine failed to achieve statistical significance. There was lack of power in these analyses, with may lead to a high degree of false-negative results. Second, the literature used for this meta-analysis originated from cross-sectional studies, which could not make causality inference. Finally, some factors that may influence catecholamine concentrations such as BMI (body-mass index), smoking, drinking, physical activity, blood pressure, were not measured or adjusted in the original studies.

## Conclusion

In conclusion, despite the above limitations, the results from our study provide a convincing evidence of the relationship between norepinephrine levels and PTSD. The evidence from this meta-analysis supports significantly higher norepinephrine levels in people with PTSD than in the controls, but no difference was found in dopamine and epinephrine concentrations between the two groups. Elevated norepinephrine levels may be an important indicator for PTSD, although whether norepinephrine could be used as a diagnostic tool requires further research.

## Author Contributions

XP and AL contributed to the study design, while XP and AK contributed to the data collection. Statistical analyses and interpretation of results were performed by XP and AK, while AL and SW drafted the manuscript and edited the language. All the authors participated in the critical revision, and approved the final version of the manuscript.

### Conflict of Interest Statement

The authors declare that the research was conducted in the absence of any commercial or financial relationships that could be construed as a potential conflict of interest.
