# Supplementary material for: Catecholamines in Post-traumatic Stress Disorder: A Systematic Review and Meta-Analysis
Source: Front Mol Neurosci. 2018 Dec 4;11:450. doi: 10.3389/fnmol.2018.00450 (PMC6288600; doi:10.3389/fnmol.2018.00450)
Supplement: Supplementary file 1 [file Data_Sheet_1.doc]

Search strategies: details of search strategy.

(Catecholamine[Title/Abstract] OR Catecholamines[Title/Abstract] OR Dopamine[Title/Abstract] OR Dopamin[Title/Abstract] OR Epinephrine[Title/Abstract] OR Norepinephrine[Title/Abstract] OR Noradrenaline[Title/Abstract] OR Noradrenalin[Title/Abstract] OR Adrenaline[Title/Abstract] OR Adrenalin[Title/Abstract]) AND (PTSD[Title] OR post-traumatic stress disorder[Title] OR posttraumatic stress disorder[Title])

220 of PubMed

TOPIC: (Catecholamine OR Catecholamines OR Dopamine OR Dopamin OR Epinephrine OR Norepinephrine OR Noradrenaline OR Noradrenalin OR Adrenaline OR Adrenalin) AND TITLE: (PTSD OR post-traumatic stress disorder OR posttraumatic stress disorder)

490 of Web of Science

('catecholamine':ab,ti OR 'catecholamines':ab,ti OR 'dopamine':ab,ti OR 'dopamin':ab,ti OR 'epinephrine':ab,ti OR 'norepinephrine':ab,ti OR 'noradrenaline':ab,ti OR 'noradrenalin':ab,ti OR 'adrenaline':ab,ti OR 'adrenalin':ab,ti) AND ('ptsd':ab,ti OR 'post-traumatic stress disorder':ab,ti OR 'posttraumatic stress disorder':ab,ti)

635 of Embase

AB ( Catecholamine OR Catecholamines OR Dopamine OR Dopamin OR Epinephrine OR Norepinephrine OR Noradrenaline OR Noradrenalin OR Adrenaline OR Adrenalin ) AND AB ( PTSD OR post-traumatic stress disorder OR posttraumatic stress disorder )

43 of PsycARTICLES
